# Effects of Maternal Fish Oil and/or 5-Methyl-Tetrahydrofolate Supplementation during Pregnancy on Offspring Brain Resting-State at 10 Years Old: A Follow-Up Study from the NUHEAL Randomized Controlled Trial

**DOI:** 10.3390/nu12092701

**Published:** 2020-09-04

**Authors:** Hatim Azaryah, Juan Verdejo-Román, Cristina Martin-Pérez, José Antonio García-Santos, Cristina Martínez-Zaldívar, Francisco J. Torres-Espínola, Daniel Campos, Berthold Koletzko, Miguel Pérez-García, Andrés Catena, Cristina Campoy

**Affiliations:** 1 Department of Paediatrics, School of Medicine, University of Granada, Avda. Investigación 11, 18016 Granada, Spain; rifappstudio@gmail.com (H.A.); joseantonio_gsantos@outlook.es (J.A.G.-S.); mcriszald@ugr.es (C.M.-Z.); fjtespinola@yahoo.es (F.J.T.-E.); dconsu@ugr.es (D.C.); 2 EURISTIKOS Excellence Centre for Paediatric Research, Biomedical Research Centre, University of Granada, 18016 Granada, Spain; 3 Mind, Brain and Behaviour International Research Centre (CIMCYC), University of Granada, 18011 Granada, Spain; j.verdejo@gmail.com (J.V.-R.); crmartinperez@gmail.com (C.M.-P.); mperezg@ugr.es (M.P.-G.); acatena@ugr.es (A.C.); 4 Instituto de Investigación Biosanitaria de Granada (Ibs-GRANADA), Health Sciences Technological Park, 18012 Granada, Spain; 5 Ludwig-Maximiliams-Universität München, Dr. von Hauner Children’s Hospital, University of Munich Hospitals, 80337 Munich, Germany; Berthold.Koletzko@med.uni-muenchen.de; 6 Spanish Network of Biomedical Research in Epidemiology and Public Health (CIBERESP), Granada’s Node, Institute of Health Carlos III, 28029 Madrid, Spain

**Keywords:** fish oil, folate, pregnancy, brain function, resting-state functional neuroimaging, neurodevelopment, children

## Abstract

Recent studies have shown that maternal supplementation with folate and long-chain polyunsaturated fatty acids (LC-PUFAs) during pregnancy may affect children’s brain development. We aimed at examining the potential long-term effect of maternal supplementation with fish oil (FO) and/or 5-methyl-tetrahydrofolate (5-MTHF) on the brain functionality of offspring at the age of 9.5–10 years. The current study was conducted as a follow-up of the Spanish participants belonging to the Nutraceuticals for a Healthier Life (NUHEAL) project; 57 children were divided into groups according to mother’s supplementation and assessed through functional magnetic resonance imaging (fMRI) scanning and neurodevelopment testing. Independent component analysis and double regression methods were implemented to investigate plausible associations. Children born to mothers supplemented with FO (FO and FO + 5-MTHF groups, *n* = 33) showed weaker functional connectivity in the default mode (DM) (angular gyrus), the sensorimotor (SM) (motor and somatosensory cortices) and the fronto-parietal (FP) (angular gyrus) networks compared to the No-FO group (placebo and 5-MTHF groups, *n* = 24) (P_FWE_ < 0.05). Furthermore, no differences were found regarding the neuropsychological tests, except for a trend of better results in an object recall (memory) test. Considering the No-FO group, the aforementioned networks were associated negatively with attention and speed-processing functions. Mother’s FO supplementation during pregnancy seems to be able to shape resting-state network functioning in their children at school age and appears to produce long-term effects on children´s cognitive processing.

## 1. Introduction

The key role of nutrition during the “first 1000 days” (from conception to 2 years after birth) on optimal brain development in later life is well established. Rather than a homogenous organ, the human brain is formed by distinct regions and processes, each of which differs in its developmental trajectory and nutritional needs. In this line, fetal life has been established as a “critical” timeframe where deficiencies of some nutrients may lead to long-lasting or irreversible effects for later neurodevelopment in offspring [1].

Maternal intake during pregnancy of certain nutrients, including long-chain polyunsaturated fatty acids (LC-PUFAs), folic acid, and other B-group vitamins, is needed for fetal nervous system development, but also for the normal development of specific brain regions (i.e., the hippocampus, striatum, retina or cortex) and to establish the neural architecture and its underlying circuitry (synaptogenesis, neuronal pruning, and myelination) that supports major brain functions [2,3,4,5].

In fact, the links between LC-PUFAs (contained in fish oil) and the function of these networks underpinning cognition in children are well established. DHA supplementation has been associated with cortical circuit maturation in children [6] and increased pre-frontal activation during sustained attention [7]. Low DHA concentrations have been associated with anomalies such as reduced indices in cortical integrity in the anterior cingulate (which implies a slower reaction time during sustained attention) [8], and increased risk for developing affective or bipolar disorders [9]. Regarding folic acid, there are consistent indications that prenatal supplements during the first 12 weeks of pregnancy not only prevent neural tube defects [10], but also affect apoptosis, neurogenesis, and overall nervous system development [11,12]. Cerebral 5-methyl-tetrahydrofolate (5-MTHF) deficiency could be associated with disturbed folate transportation or increased turnover from the central nervous system, which could lead to atrophy of fronto-temporal regions and periventricular demyelination, as can be observed in some neuroimaging studies [13]. 

Resting-state functional magnetic resonance imaging (rsfMRI) has been identified as a useful tool to explore brain function in the absence of task-demanding stimuli [14]. The rsfMRI allows for identification of the intrinsic brain networks implicated in primary (i.e., sensorimotor or visual networks) and high-order functions (i.e., fronto-parietal or default mode networks), such as executive functions or attention networks [15]. Despite the relevance of nutritional supplementation on brain structure, little is known about the effects of these nutrients on brain functioning in humans. A previous study has shown associations between PUFAs and the functional connectivity in areas within the default mode network, and the visual and limbic systems in elderly adults [16]. 

Recently, in a previous publication from the Nutraceuticals for a Healthier Life (NUHEAL) project, a long-term early nutrition programming effect of maternal folate and omega-3 fatty acid supplementation on offspring metabolism has been suggested [17]. To the best of our knowledge, no other study has explored the early nutrition programming effects on brain resting-state in school children at 10 years old. Due to the lack of research in this field, in the present study, we hypothesized that maternal prenatal supplementation with fish oil (FO) and/or 5-methyl-tetrahydrofolate (5-MTHF) might have a long-term effect on the brain resting-state networks of offspring at the age of 9.5–10 years. Furthermore, we will also explore the association between functional connectivity and children’s mental processing.

## 2. Materials and Methods

### 2.1. Study Design

The current study was conducted as a follow-up of the Spanish children enrolled in the NUHEAL double-blind randomized trial www.ClinicalTrials.gov Identifier NCT01180933). A detailed study design, subject recruitment, and population characteristics have been described elsewhere [18]. Briefly, NUHEAL is a multicenter, randomized, double-blind, 2 × 2 factorial placebo-controlled trial designed to assess the effects of *n*-3 LC-PUFAs and 5-MTHF supplementation during the second half of pregnancy on infant development. Healthy pregnant women were recruited before the 20th weeks of gestation and were randomly assigned to four different groups. From week 20 to delivery, they received a daily supplement consisting of fish oil (FO: 500 mg of docosahexaenoic acid (DHA) + 150 mg eicosapentaenoic acid (EPA)), 400 μg 5-MTHF, both, or placebo, together with vitamins and minerals following European recommendations for pregnant women. All children were born at term and with birth weight appropriate for gestational age. At the age of 9 years, a total of 154 NUHEAL children were assessed by the NUTRIMENTHE neuropsychological battery (NNB) [19], and 85 of them also underwent an MR-scanning session at the age of 9.5–10 years. 

The study was approved by the Ethics Committees for human research of the Universidad de Granada (Grant agreement number: 212652) and conducted in accordance with the Helsinki Declaration for human research studies in 2013 [20]. Written informed consent forms were obtained from all participants at the beginning of the study and before the magnetic resonance session.

### 2.2. Sample Size

**Figure 1:**
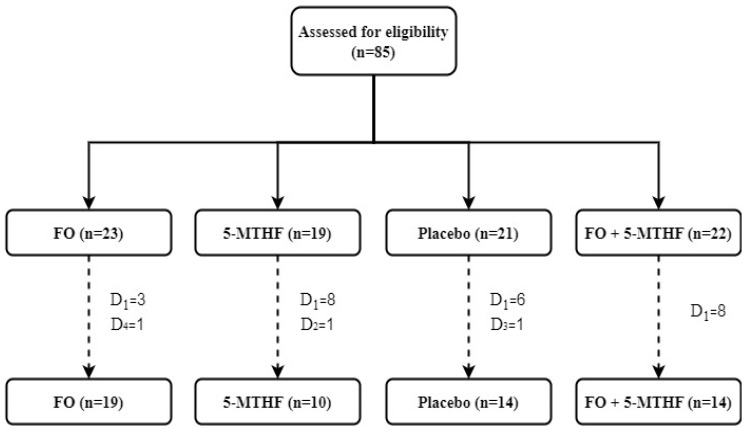
Participant flowchart. FO = fish oil, 5-MTHF = 5-methyl-tetrahydrofolate. Discards, D_1_ = excessive motion, D_2_ = previous head injuries, D_3_ = claustrophobia, D_4_ = implanted ferromagnetic object.

A total of 85 Spanish children underwent an MR-scanning session at the age of 9.5–10 years. Twenty-eight of the datasets were discarded due to excessive motion, previous head injuries, claustrophobia and implanted ferromagnetic objects; therefore, a total of 57 children had valid data for all NNB and resting-state functional magnetic resonance imaging (rsfMRI) and were included in the analyses. From these children, 19 were born to mothers who received FO, 10 whose mothers were supplemented with 5-MTHF, 14 whose mothers received placebo and 14 were born to mothers supplemented with FO + 5-MTHF during pregnancy (Figure 1). All participants met the inclusion and exclusion criteria established for this study.

To achieve a statistical power of 80%, a medium-to-large effect size (0.7–0.9) between study groups, and considering a significance level as 0.05, the minimum sample size required is 20 per group [21]. Due to a high number of discards, future clustering of our originals groups will be made to reach the minimum sample size required for a robust and reliable neuroimaging analysis.

### 2.3. Resting-State fMRI Experimental Procedure (Primary Outcome)

Before starting the fMRI acquisition, children were trained by a master’s degree psychologist to lie down on the patient table, keep calm and close their eyes during the scanner session. They were instructed to think about nothing in particular, but not to fall asleep during the resting-state acquisition. Resting-state brain images were acquired for a total time of 6 min.

#### 2.3.1. Independent Component Analyses

Independent component analyses (ICA) was carried out using the probabilistic independent component analysis [22] as implemented in MELODIC (Multivariate Exploratory Linear Decomposition into Independent Components) version 3.14, part of FSL (FMRIB Software Library). Pre-processed data were whitened and projected onto a 40-dimensional subspace using probabilistic principal component analysis. The forty components were visually checked and identified. Components that represent well-known artifacts as motion, high-frequency noise, or venous pulsation [23,24] or those not located mainly in the gray matter [25] were identified and not taken into account in the following analyses. Fourteen components were discarded because of the previous reason; and the remaining twenty-six resting-state networks (RSNs) were labeled based on the overlap with the Harvard–Oxford cortical and subcortical structural atlases available in FSL. 

#### 2.3.2. Imaging Data Acquisition and Pre-Processing

fMRI data were collected with a 3T Philips Intera Achieva System (Philips Medical Systems, Eindhoven, The Netherlands) equipped with an eight-channel phased-array head coil. A T2*-weighted echo-planar imaging (EPI) sequence was acquired with the following parameters: repetition time (TR) = 2000 ms; echo time (TE) = 30 ms; flip angle = 78°; field of view = 230 × 230 mm; number of slices = 23; voxel dimension = 3 × 3 × 4 mm; gap, 1 mm. Structural images were also obtained as an isotropic T1-weighted turbo-gradient-echo sequence in the sagittal plane (TR = 8.3 ms; TE = 3.8 ms; flip angle = 8°; FOV (Field Of View) = 240 × 240 mm; number of slices = 160; slice thickness = 1 mm; voxel dimension = 1 × 1 × 1 mm). Prior to specific resting-state preprocessing, motion during acquisition was estimated using the realign tool implemented in statistical parametric mapping (SPM8) software (Wellcome Department of Cognitive Neurology, Institute of Neurology, Queen Square, London, UK). Subjects with motion parameters exceeding 3 mm or 3 degrees were excluded from the subsequent processing and analyses.

Resting-state functional imaging pre-processing was performed using Functional MRI of the Brain (FMRIB) Software Library (v5.0.9, FSL, http://fsl.fmrib.ox.ac.uk/fsl/fslwiki). Pre-processing steps included the removal of the ten first volumes, high-pass temporal filtering (120s), motion correction using the MCFLIRT tool [26], brain extraction using BET (Smith, 200), spatial smoothing using a Gaussian kernel of FWHM (Full Width at Half Maximum) = 8 mm, registration to a T1-weighted standard template using the FSL linear registration tool (FLIRT) with 12 degrees of freedom (DOF) and finally resampling to a 4 mm resolution.

### 2.4. Neuropsychological Assessment (Secondary Outcome)

Children´s neurocognitive development was assessed using a NUTRIMENTHE neuropsychological battery (NNB), a comprehensive neuropsychological battery specifically developed for the NUTRIMENTHE project [19,27,28]. For the present study, NNB was used to evaluate long-term effects of prenatal supplementation on the whole spectrum of neuropsychological functioning in children. The NNB consists of fifteen cognitive tests to assess seven different neuropsychological domains: processing speed, perception, motor, memory, attention, language, and executive functions, which are described briefly in Table 1 (a full description was already published elsewhere [19]).

### 2.5. Statistical Analyses

#### 2.5.1. Resting-State fMRI

A dual regression method, described elsewhere [30], was implemented in FSL, followed by two-group *t*-tests, to compare brain maps across groups. Briefly, subject-specific statistical brain maps were created and collapsed in a 4D file for each resting-state network. In the first stage of the dual regression method, the spatial components generated during the ICA analysis were regressed into each subject’s resting-state data to give a set of subject-specific time courses for each component. In the second stage, those time courses were regressed into the resting-state data to obtain subject-specific spatial brain maps for each component. Then, those subject-specific brain maps were used to compare each brain network between groups. These comparisons were tested voxel-wise for differences between groups using nonparametric permutation testing (5000 permutations) [31]. For each RSN, the resulting statistical maps had a threshold at *p* < 0.05 (threshold-free cluster enhancement-corrected for family-wise errors). 

#### 2.5.2. Neuropsychological Outcomes

The normality of variables was assessed for both RSN scores and neurodevelopment outcomes with Shapiro–Wilk’s method. NNB outcomes were represented as mean (standard errors), and significant differences were assessed through Student *t* and Kruskal–Wallis tests. Pearson and Spearman correlations between RSN scores and NNB tests were carried out, taking into account the normality of variables. 

## 3. Results

The general characteristics of the study population obtained at the study entry before the fMRI session are shown in Table 2. 

### 3.1. Resting-State fMRI Results

**Figure 2:**
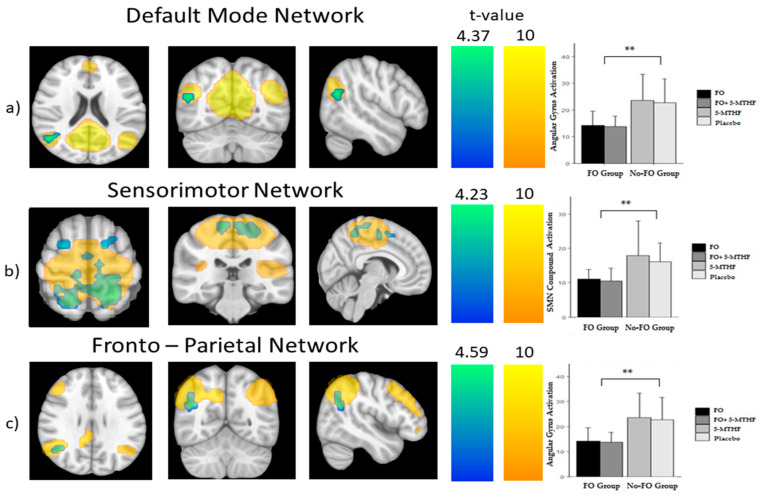
Children’s resting-state functional magnetic resonance imaging (fMRI). Voxel-wise significant differences between groups according to maternal supplementation or not with FO during pregnancy. Color bars represent t-value. Warm colors represent the whole brain network, whereas cold colors represented over the networks show the brain areas with significant differences between groups. (**a**) Default mode network, significant area: angular gyrus; (**b**) sensorimotor network, significant areas: postcentral gyrus, precentral (left and right) gyrus and putamen; (**c**) fronto-parietal network, significant area: angular gyrus. (FO: fish oil, 5-MTHF: 5-methyl-tetrahydrofolate). ** P_FWE_ < 0.05.

To assess the effect of prenatal FO supplementation, participants were clustered into two groups: (i) 33 children from the FO group (FO or FO + 5-MTHF), and (ii) 24 children from the No-FO group (5-MTHF alone or placebo). Three resting-state networks showed significant differences between the study groups. Specifically, children from No-FO group showed strong functional connectivity in the default mode (DM) (angular gyrus), the sensorimotor (SM) (motor and somatosensory cortices) and the fronto-parietal (FP) (angular gyrus) networks compared to the FO group (P_FWE_ < 0.05) (see Figure 2 and Table 3).

Alternatively, to assess the effect of folate supplementation during pregnancy, participants were divided into the following: (i) 24 children born to mothers supplemented with 5-MTHF or FO + 5-MTHF and (ii) 33 children whose mothers were supplemented during pregnancy only with FO or placebo. We found no significant differences in brain networks during resting-state between children supplemented with or without 5-MTHF.

### 3.2. Neuropsychological Outcomes and RSN Scores

No significant differences were found between the FO and the No-FO groups regarding the neuropsychological test results (see Table 4), except for a trend of children belonging to the FO group performing better in the Recall of Objects (ROT) test (memory domain) (*p* = 0.065). 

**Figure 3:**
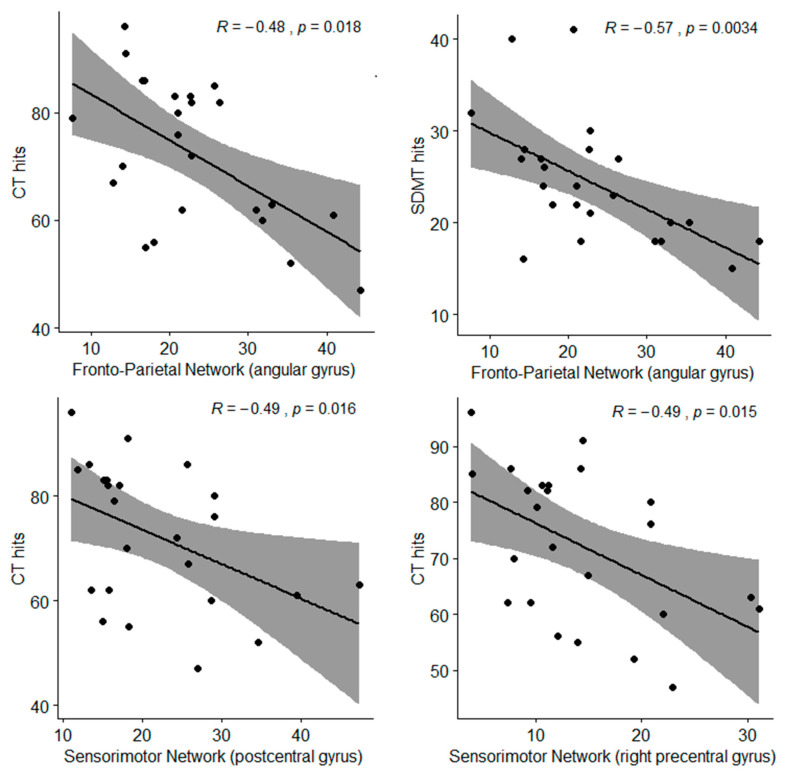
Spearman correlations between NNB and RSN scores; *R* = correlation coefficient; *p* = *p*-value; CT = Cancellation Test (total hits) from the attention domain; SDMT = Symbol Digit Modalities Test (total hits) from the speed-processing domain.

In Table 5, correlations established between RSN scores and neuropsychological results are shown, considering the FO or No-FO groups. No significant results were found between the default mode network (DMN) and the NNB scores. The No-FO group showed a negative association between the angular gyrus within the fronto-parietal network (FPN) regarding SDMT hits (speed-processing domain) and CT hits (attention domain) (See Figure 3). In addition, the No-FO group presented a negative correlation between the precentral (right) and postcentral gyrus within the SMN (sensorimotor network) and CT hits (attention domain) (See Figure 3). 

## 4. Discussion

This is the first study to examine the long-term effect of maternal supplementation with FO and/or 5-MTHF on the RSNs of the offspring at school age. We found that FO, but not 5-MTHF, supplementation during the second half of pregnancy is associated with decreased functional connectivity of children’s brain networks at 9.5–10 years of age. Specifically, the default mode, the sensorimotor and the fronto-parietal networks displayed weaker functional connectivity in children born to mothers supplemented with FO or FO + 5-MTHF. Furthermore, after correlating the resting-state scores and NNB tests, we found that children born to mothers who did not take FO supplements performed poorly regarding speed processing and attention tests. 

Weak functional connectivity does not necessarily indicate poor cognitive neurodevelopment; in fact, some studies have shown for instance that the variance in IQ levels within a heterogeneous population was mostly explained by the distributed communication efficiency of brain networks built using moderately weak, long-distance connections, with only a smaller contribution of stronger connections [32]. In this study, children exposed to prenatal FO did not show any functional disadvantage but tended to have better memory. Another example is the weaker brain resting-state of bilingual subjects compared to monolingual ones [33,34], where the strength of resting-state functional connectivity correlated inversely with behavioral performance. 

Considering the main brain area differences found in the present study, the angular gyrus is a cross-modal region which might act as a “connector hub” for the global processing of information [35], but also as a shifting area between internal (DMN) and external/salient (FPN) information [36]. These functions are competing but complementary [37] and could mask a high intrinsic relationship between functional connectivity and neurodevelopment. Indeed, the children born to mothers not supplemented with FO showed that the angular gyrus activity within the FPN was negatively related to the performance in attention and processing speed, suggesting an FO-related conjoint alteration between the most relevant brain network related to cognition (FPN) and the performance in important cognitive processes. 

Regarding several alterations in the sensorimotor network (SMN), both somatosensory (postcentral) and motor (putamen and precentral gyrus) areas are involved in pre-mediated state of readiness to perform/coordinate a motor task [38], and their alterations in healthy subjects might lead to a maladaptive coordination of movements and motor learning, as proposed by previous animal studies [38]. However, this brain network´s function is not limited to motor execution. Indeed, it also mediates action execution programs [39], and its activity is known to be more related to attention to external stimuli in comparison with internal attentional processes [40]. Those children born to mothers not supplemented with FO showed a negative correlation between SMN and the performance in neuropsychological tasks related to attention; these results support the previously studied association between attentional and speed-processing processes and the SMN function [40]. It is well established that motor performance, visual-motor coordination [41] and attention [3] start to develop during gestation, especially at the second and third trimester of pregnancy; thus, these domains may be affected by nutritional interventions during this critical period of neurodevelopment, noting that omega-3 polyunsaturated fatty acids are essential due to their role in neurogenesis, fluidity and membrane fatty acid composition [42].

To date, clinical literature focused on neurodevelopmental effects of key nutrients is broadly based on nutrient deficiencies or deprivation, but optimal nutrient doses and potential long-term effects at different developmental ages remain unclear [1,43]. Some studies suggest that prenatal or postnatal dietary interventions based on LC-PUFAs could have long-term effects on late child neurodevelopment, mainly on sustained attention, language, and processing speed [44,45,46,47]. For instance, children whose mothers received fish oil supplementation performed significantly better than the placebo group regarding the communication domain at the ages of 4 and 6 months [48], eye and hand coordination at 2.5 years [49], and sustained attention at 5 years [44]. However, there is no conclusive RCT evidencing long-term benefits of LC-PUFA supplementation during early life on neurodevelopment in healthy term infants [50,51,52], reflecting differences in terms of timing of supplementation, doses, and combination with other micronutrients. In relation to folic acid, some epidemiological studies [53] and a previous EEG/ERP (Electroencephalography/Event-Related Potential) research from the NUHEAL project [54] have reported that prenatal folate supplementation improves neurodevelopmental performance (mainly in attention system) in offspring at school age. However, there is no clear or robust evidence to support the use of multivitamin-containing folic acid supplementation during pregnancy on mental performance later in life [55,56]. Interestingly, these contradictory results come from the evaluation of mental performance as a global measure or an inappropriate timeframe for targeted neuropsychological domains, which could mask specific effects of a nutritional intervention during “critical periods” of brain development on long-term neurodevelopment. We must keep in mind that our No-FO group comprehends not only the 5-MTHF subgroup, but also the placebo subgroup, and the results of this study are directed to enforce the protective role of maternal LC-PUFA supplementation on children’s brain development and function.

The main strengths of this study are in relation to its long term follow-up and monitoring, brain imaging to assess functional connectivity, and the use of an extensive neuropsychological batteries of tests to measure different brain domains. This novel approach has facilitated the study of associations between early nutrition, brain networks, and mental performance. The results provided in this manuscript provide more insight into the impact of FO supplementation during pregnancy on children’s neurodevelopment and brain functioning. A large number of validated techniques have been used to assess our hypothesis, and the data come from a well-established cohort. 

The main limitation of this study is the relatively small sample size in combination with multiple outcomes. Due to a large number of participants being excluded from the study (32.9%), mainly due to excessive movement during the rsfMRI session, we were required to cluster our four initial groups into two. In this case, we were able to guarantee 80% of statistical power (minimum required n = 20 for each group) and to detect a medium-to-large effect size (0.7–0.9). Furthermore, to avoid false positives regarding our neuroimaging analysis, the TFCE (threshold-free cluster enhancement) is one of the most reliable approaches, which, in our case, involves 5000 permutations to control for the family-wise error rate. We believe that large differences between study groups are considerably rare and unlikely. All subjects eligible for this study are within the normal range of cognitive capabilities with no previous neuropathology diagnosed. Nevertheless, the results obtained here can be interpreted as preliminary data and cannot be considered generalizable. Further research in this area is needed to optimize the recommendations regarding LC-PUFAs and folic acid supplements during pregnancy. 

## 5. Conclusions

This study further elucidates how maternal FO supplementation during pregnancy may be able to shape the resting-state network functioning of children at school age and as a consequence to produce effects on children´s cognitive processing; these results reinforce the idea of an early nutrition programming effect on brain functioning during childhood.

## Figures and Tables

**Table 1:** NUTRIMENTHE neuropsychological battery description [19,28,29].

Domain	Function	Test
Memory	Visual Episodic Memory	Recall of Objects Test (ROT)
	Verbal Memory	Rey Auditory Verbal Learning Test (RAVLT)
Attention	Sustained and Focused Attention	Continuous Performance Test (CPT)
	Spatial Attention	Pair Cancellation Test (W-M)
Motor	Visio-Motor Coordination	Grooved Pegboard Test (GPT)
Perception	Visio-Perceptual Integration	Hooper Visual Organization Test (HVOT)
Language	Semantic Fluency	Categorical Fluency Test (F-A-S-Animals)
	Verbal Comprehension	Token Test II (NEPSY-II)
Processing Speed	Processing Speed	Symbol Digit Modalities Test (SDMT)
Executive Functions	Impulsivity/Inhibition	Stroop Color and Word Test
	Update	Reversal Digits Subtest
		Matrix Analogies Test-(K-ABC-II)
	Flexibility/Shifting	Children’s Color Trail Test (CCTT)
	Decision Making	Hungry Donkey Task (HDT)

**Table 2:** Sociodemographic characteristics of the study population by study group.

	FO (*n* = 19)	5-MTHF (*n* = 10)	Placebo (*n* = 14)	FO + 5-MTHF (*n* = 14)
Mothers’ characteristics				
Age, years	29.68 (4.73)	34.15 (5.77)	31.86 (3.25)	30.68 (4.81)
BMI (20 w), kg/m^2^	25.96 (3.67)	24.87 (2.13)	25.63 (2.47)	26.40 (2.89)
BMI (30 w), kg/m^2^	28.47 (4.19)	26.64 (2.07)	27.50 (2.50)	28.46 (2.59)
BMI (delivery), kg/m^2^	29.98 (4.58)	27.81 (2.05)	29.08 (3.08)	29.39 (2.86)
Weight gain, kg	10.64 (3.65)	7.67 (3.31)	8.86 (3.16)	7.25 (4.61)
Relative weight gain, %	15.45 (5.14)	12.08 (6.30)	13.49 (5.15)	10.99 (7.33)
Education *				
Mother	8 (42.1)	2 (50.00)	4 (50.00)	4 (44.45)
Father	11 (57.9)	2 (50.00)	4 (50.00)	5 (55.55)
Parity, *n* (%)				
0	13 (68.42)	5 (50.0)	7 (50.0)	6 (42.86)
≥1	6 (31.58)	5 (50.0)	7 (50.0)	8 (57.14)
Smoking (20 w), *n* (%)	5 (26.32)	1 (10.0)	0 (0.0)	4 (28.57)
Gravidity risk at 20 w, *n* (%)				
No risk factors	3 (15.79)	3 (30.0)	5 (38.46)	3 (21.43)
≥1 risk factor	16 (84.21)	7 (70.0)	8 (61.54)	11 (78.57)
Delivery risk				
No risk factors	6 (31.58)	4 (40.0)	7 (53.85)	5 (35.71)
≥1 risk factor	13 (68.42)	6 (60.0)	6 (46.15)	9 (64.29)
Children’s characteristics				
Age, years	9.67 (0.26)	9.76 (0.20)	9.75 (0.19)	9.69 (0.22)
BMI, kg/m^2^	19.45 (4.22)	17.64 (2.60)	17.76 (2.87)	19.08 (2.87)
Sex, *n* (%)				
Female	8 (42.11)	7 (70.0)	3 (21.43)	6 (42.86)
Male	11 (57.89)	3 (30.0)	11 (78.57)	8 (57.14)

FO: fish oil; 5-MTHF: 5-methyl-tetrahydrofolate; BMI: body mass index. * Attained general qualification level for university entrance or university degree. For qualitative variables (frequency (percentage)); for quantitative variables (mean (standard deviation)).

**Table 3:** Brain regions showing differences between groups (P_FWE_ < 0.05).

	Side	MNI Coordinates			Volume (mm^3^)	t-Value
		X	Y	Z		
Default Mode Network						
Angular Gyrus	R	50	−62	20	176	4.37
Sensorimotor Network						
Postcentral Gyrus	L	−26	−50	60	2792	3.83
Precentral Gyrus	L	−18	2	44	760	4.17
Precentral Gyrus	R	22	−2	68	672	3.40
Putamen	L	−22	10	12	336	4.23
Fronto-Parietal Network						
Angular Gyrus	R	46	−58	28	296	4.58

MNI: Montreal Neurological Institute; X, Y and Z: Axis; R: Right, L: Left.

**Table 4:** Hits, timing and scores obtained by school children in the neuropsychological assessment depending on their mothers’ fish oil supplementation during pregnancy.

NNB	FO Group (FO and FO + 5-MTHF) *n* = 33	No-FO Group (5-MTHF + Placebo) *n* = 24	*p*-Value
SDMT Hits	24.74 (0.96)	24.38 (1.38)	0.812
Grooved DH	45.03 (2.51)	40.25 (2.41)	0.162
Grooved NDH	54.77 (4.87)	45.17 (3.42)	0.101
HVOT Hits	16.06 (0.59)	16.62 (0.61)	0.512
CT Hits	74.12 (2.7)	72.33 (2.76)	0.654
CPT BL7 OMI	9.35 (1.43)	9.25 (1.39)	0.962
ROT Immediate Hits	5.81 (0.4)	4.83 (0.34)	0.065
ROT Delayed Hits	0.34 (0.15)	0.25 (0.09)	0.589
RAVLT Hits Trial 1	4.61 (0.25)	4.5 (0.42)	0.795
RAVLT Hits Trial 1–5	10.29 (0.35)	9.71 (0.49)	0.344
RAVLT Delayed Trial	13.13 (0.3)	12.83 (0.39)	0.562
Animals Total Hits	12.38 (0.63)	12.5 (0.65)	0.875
Token Test Total Hits	20.78 (0.37)	21.58 (0.58)	0.255
Stroop Interference	−1.57 (0.87)	−1.47 (1.18)	0.514
Reversal Digits, Hits	9.12 (0.41)	9.46 (0.63)	0.652
K-ABC-II	12.75 (0.46)	13.46 (0.88)	0.451
CCTT Part 1(s)	107.94 (5.8)	108.71 (10.13)	0.352
HDT Total Hits	0.81 (3.96)	0.27 (3.45)	0.114

NNB: NUTRIMENTHE neuropsychological battery; SDMT, Symbol Digit Modalities Test (total hits); DH, dominant hand; NDH, non-dominant hand; HVOT, Hooper Visual Organization Test (total hits); CT, Cancellation Test (total hits); CPT BL7, continuous performance test (total hits), Block7; OMI, omissions; ROT, Recall of Objects test (immediate and delayed recalled pictures); RAVLT, Rey Auditory Verbal Learning Test (recalled words in trial 1, trial 1–5 and delayed (hits)); K-ABC-II, Matrix Analogies Test (total hits); CCTT, Children’s Color Trail Test (time, part 1(s)); HDT, Hungry Donkey Task (total score); (s), seconds. FO: fish oil; 5-MTHF: 5-methyl-tetrahydrofolate. Data are expressed by mean (standard errors). *p*-Value from Student *t*-test or Kruskal–Wallis test depending on normality.

**Table 5:** Correlations between resting-state network (RSN) scores and neuropsychological battery (NNB) tests for each supplementation group.

NNB	Default Mode Network		Sensorimotor Network								Fronto-Parietal Network	
	Angular Gyrus		Postcentral Gyrus		Precentral Gyrus (L)		Precentral Gyrus (R)		Putamen		Angular Gyrus	
	FO	No-FO	FO	No-FO	FO	No-FO	FO	No-FO	FO	No-FO	FO	No-FO
SDMT hits ^s^	0.055	−0.110	0.180	−0.245	0.130	−0.300	−0.005	−0.225	0.147	−0.211	−0.013	−0.570 *
Grooved DH ^s^	−0.144	0.046	−0.244	0.210	−0.114	0.411	0.160	0.350	0.044	0.250	−0.299	0.162
Grooved NDH ^s^	0.043	−0.079	−0.349	0.082	−0.189	0.240	−0.197	0.183	0.066	0.161	−0.280	0.223
HVOT Hits ^s^	−0.044	0.092	0.221	−0.033	−0.077	−0.236	−0.224	−0.001	−0.154	−0.056	0.165	0.182
CT Hits ^s^	−0.147	−0.332	0.040	−0.489 *	0.109	−0.228	−0.26	−0.491 *	−0.042	−0.164	−0.283	−0.481 *
CPT BL7 OMI ^s^	0.106	−0.137	0.201	−0.044	0.214	0.063	0.355	−0.05	0.144	−0.221	0.085	−0.073
ROT Immediate Hits ^s^	0.219	−0.138	0.237	−0.231	−0.114	−0.274	−0.067	−0.282	−0.224	0.026	−0.060	−0.362
ROT Delayed Hits ^s^	0.084	−0.108	−0.077	−0.152	0.470	−0.026	0.095	−0.219	0.180	−0.167	0.427	−0.285
RAVLT Hits Trial−1 ^p^	−0.285	−0.206	−0.076	−0.136	0.007	−0.108	−0.025	−0.159	−0.379	0.162	0.055	−0.187
RAVLT Hits Trial-1-5 ^s^	−0.166	−0.031	0.084	−0.042	−0.111	−0.021	0.425	−0.095	−0.497	0.175	0.161	−0.324
RAVLT Delayed Trial ^s^	0.145	−0.233	−0.043	0.138	0.022	0.228	−0.198	0.155	−0.121	0.357	0.077	0.029
Animals Total Hits ^s^	0.072	−0.209	−0.271	−0.206	0.112	0.151	−0.503	−0.147	−0.019	0.439	−0.105	−0.371
Token Test Total Hits ^p^	−0.037	−0.190	0.163	−0.153	−0.312	−0.222	−0.161	−0.069	−0.045	0.04	−0.191	−0.126
Stroop Interference ^p^	0.267	−0.253	0.369	−0.315	0.042	−0.394	0.017	−0.200	0.120	−0.247	0.002	0.047
Reversal Digits, Hits ^p^	−0.221	0.139	−0.081	−0.038	−0.108	−0.188	−0.149	−0.118	−0.165	0.011	−0.520	−0.136
K-ABC-II ^s^	0.124	−0.276	0.124	−0.221	−0.035	−0.187	−0.147	−0.302	0.201	−0.160	−0.196	−0.255
CCTT Part 1 (sec) ^p^	0.087	0.119	0.101	0.155	−0.214	0.003	0.054	0.339	0.292	0.005	0.137	−0.214
HDT Total Hits ^s^	−0.041	0.084	0.259	0.232	0.017	0.122	0.478	0.258	0.214	0.283	−0.008	0.328

NNB: NUTRIMENTHE neuropsychological battery; SDMT, Symbol Digit Modalities Test(total hits); DH, dominant hand; NDH, non-dominant hand; HVOT, Hooper Visual Organization Test (total hits); CT, Cancellation Test (total hits); CPT BL7, continuous performance test (total hits), Block7; OMI, omissions; ROT, Recall of Objects test (immediate and delayed recalled pictures); RAVLT, Rey Auditory Verbal Learning Test (recalled words in trial 1, trial 1–5 and delayed (hits)); K-ABC-II, Matrix Analogies Test (total hits); CCTT, Children’s Color Trail Test (time, part 1 (s)); HDT, Hungry Donkey Task (total score); (s), seconds; FO = fish oil. Data are expressed as r correlation coefficient from p = Pearson or s = Spearman, * *p* < 0.05.

## Abbreviations

5-MTHF	5-methyl-tetrahydrofolate
CCTT	Children’s Colors Trail Test
CPTBL7	Continuous Performance Test Block7
CT	Cancellation Test
DHA	Docosahexanoic acid
DH	Dominant Hand
DMN	Default Mode Network
EPA	Eicosapentaenoic acid
FO	Fish Oil
FPN	Fronto-Parietal Network
HDT	Hungry Donkey Task
HVOT	Hooper Visual Organization Test
K-ABC-II	Matrix Analogies Test
LC-PUFAs	long-chain polyunsaturated fatty acids
NDH	Non Dominant Hand
NNB	NUTRIMENTHE Neuropsychological Battery
NUHEAL	NUtraceuticals for a HEALthier life
RAVLT	Rey Auditory Verbal Learning Test
ROT	Recall of Object Test
rsfMRI	Resting-state functional magnetic resonance imaging
RSN	Resting-State Network
SDMT	Symbol Digit Modalities Test
SMN	SensoriMotor Network
